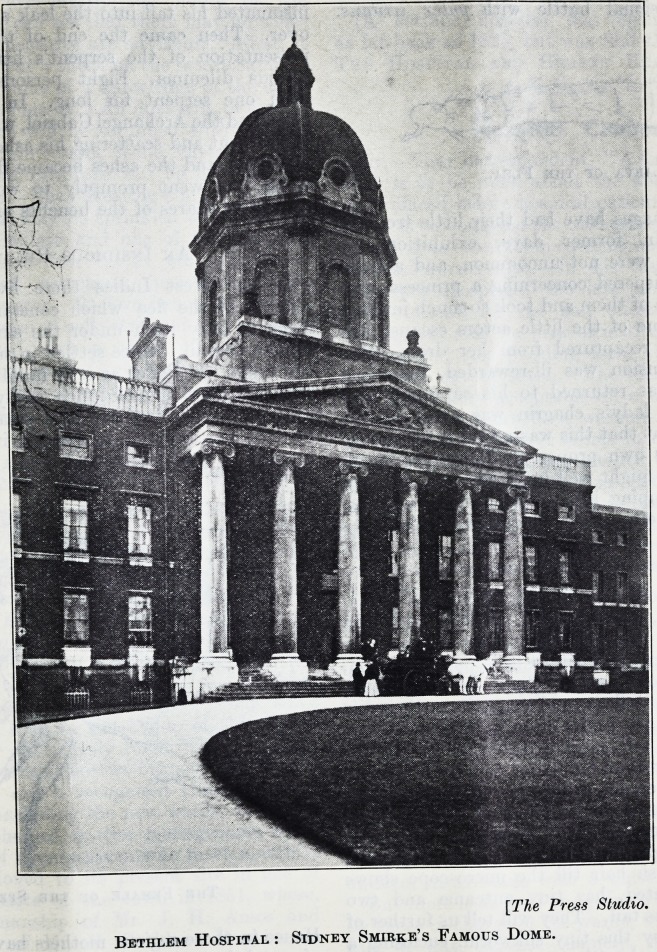# The Removel of Bethlem Hospital

**Published:** 1924-11

**Authors:** 


### THE REMOVAL OF BETHLEM HOSPITAL.

The news that the Corporation of the City of London are considering a scheme for the removal of Bethlem Hospital does not come as a surprise. The rather dreary and confined surroundings of Lambeth can certainly not supply that atmosphere of hope and cheerfulness which is now regarded as so important in the treatment of the insane, and there is comparatively little scope for outdoor exercise and occupation. The contemplated removal will not be the first time that Bethlem has changed its quarters. The original "Bedlam" was situated on ground now occupied by Liverpool Street Station, and from there it was moved to Moorfields. Later, when the buildings grew too depressing and unsuitable, came the change to Lambeth, which in 1812 was still quiet and rural.

The dome of Bethlem has been a familiar sight to dwellers on the south side of the river, and thousands of afflicted people have found healing beneath its shade. But an institution planned so long ago, in a period when the care of the insane was in an elementary state, is obviously unlikely to meet in every respect the requirements of to-day. Moreover, a change of site might help to free the hospital of a heavy deficit. More than half its patients were last year treated free of all charge; others paid only relatively small amounts. Nor are the arrangements for the nurses altogether adequate, and where work is so trying and calls for so much skill and patience it is only fair that the staff should be made as comfortable as modern convenience can compass. Bethlem is intended for curable persons, and it receives out-patients as well as in-patients, and for the latter a convalescent home in the country is provided. Everything possible has been done to bring the hospital up to date, but it would appear that the limit of what can be accomplished has now been reached. In the interest of its out-patients it would seem desirable that the move should not be too far away, and the choice of a site may be difficult. But it is abundantly clear that the troubled and clouded mind is more likely to grow clear and strong again among the sights and sounds of the country than in the noise and airlessness of London. Trees and flowers are more inspiring than smoke and chimney-pots, and the proposal to remove Bethlem is one that must have the support of all who, recognising the peculiarly sad lot of the mentally unsound, are anxious for them to have every chance to regain normality.

[*The Press Studio.*

BETHLEM HOSPITAL: SIDNEY SMIRKE'S FAMOUS DOME.

Mental normality is, we are at length beginning to realise, closely allied to physical normality, and a patient in an asylum is just as much in need of air and sunshine as a patient in a sanatorium for the tuberculous. It is also particularly desirable that he should not be ceaselessly reminded of his infirmity by a multitude of little restrictions, and it is easier to give him some measure of freedom in country than in town. The old-fashioned asylum will soon happily be a thing of the past, and it is in accordance with the fine traditions of this charity that it should be ahead of its time.

## Figures and Tables

**Figure f1:**